# The rare disease challenge and how to promote a productive rare disease community: Case study of Birt-Hogg-Dubé Symposia

**DOI:** 10.1186/1750-1172-7-63

**Published:** 2012-09-05

**Authors:** Vicki L Colledge, John Solly

**Affiliations:** 1 Myrovlytis Trust, 26 Cadogan Square, London, SW1X 0JP, UK

**Keywords:** Rare disease, Symposia, Birt-Hogg-Dubé, BHD, Renal cell carcinoma

## Abstract

Resources for rare diseases are lacking. Patients do not have the information and support that they need, and researchers struggle to make progress due to a shortage of skills and collaborations within the field. One way to overcome these hurdles is to host annual Symposia, focused on a specific rare disease. Here, we use the example of Birt-Hogg-Dubé Symposia to discuss the practical issues of such meetings, including the importance of timing and the choice of invited speakers. We highlight the ways in which rare disease symposia can create a single community, removing barriers between patients, clinicians and researchers.

## Letters to the editor

There are estimated to be around 7,000 rare diseases, with 6% of the global population affected by one at some stage in their life [1]. Despite the high number of people affected by rare diseases, resources are lacking. Patients often feel isolated, unable to get the information and support they need. With so few researchers working in the rare disease field, support, skills and collaborations can also be lacking. This inefficient method of research hinders our understanding of rare diseases and slows the development of treatments.

With the necessary skills and resources missing, how can we advance rare disease research and support? One method is to host scientific and patient meetings. Scientific meetings are an excellent opportunity for researchers working in a similar field to come together, learn about recent advances in the area, foster new collaborations and stimulate new ideas. Here, we discuss the ways in which such symposia can contribute to and increase the pace of rare disease research, by using the example of the BHD Symposia, an annual global meeting organized by the Myrovlytis Trust.

Birt-Hogg-Dubé (BHD) Syndrome is a rare genetic disorder characterized by benign skin tumors, lung cysts leading to pneumothorax and an increased risk for renal cell carcinoma [2,3]. Patients can develop any combination of the symptoms, making diagnosis difficult and meaning that BHD is probably under diagnosed [4]. As such, it is important to raise awareness of BHD among patients and clinicians. The Myrovlytis Trust, a medical charity focusing on rare genetic diseases, funds research into BHD syndrome and organizes annual symposia, uniting the BHD community. Four International BHD Symposia have taken place since 2008, bringing together researchers, clinicians and patients. Each Symposium has grown in the number of delegates, with the Fourth BHD Symposium, held in March 2012, hosting over 90 attendees, about a third of whom were patients and family members.

As with all scientific meetings, sharing unpublished data and discussing ideas is key to making rapid progress in the field. However, for rare diseases, the importance of symposia extends beyond that of standard scientific meetings. With so few individuals studying the disease, symposia are important for generating a community feel. Researchers are often geographically isolated and find it difficult to form strong collaborations. Symposia are the perfect opportunity for scientists from across disciplines to meet and share their skills.

BHD researchers are located all across the world, but often with only one or two institutions in each country involved in BHD research. BHD Symposia mitigate the feeling of isolation for these researchers, and provide sources of help and motivation. The Symposia also allow BHD researchers to meet, discuss challenges and ideas and form new collaborations.

As many BHD patients do not know anyone else with BHD outside their own family, it is valuable to bring patients together to share stories, ask advice and jumpstart peer support. The BHD Symposia are joint meetings for researchers and patients, with both mixed and separate sessions. The aim of the Symposia is to generate the sense of a single BHD community, removing barriers between patients, family members, scientists and clinicians.

Awareness of BHD amongst the wider research community is central to making progress in understanding the disease. Attracting new researchers to the field would bring in new skills and resources, as well as a fresh perspective on research. Awareness can be achieved through widely advertising the symposium, but also by careful selection of the invited speakers and the timing of the meeting.

As well as inviting experts from within the BHD field to speak at the BHD Symposia, other world-class researchers are also invited who could speak on a broader topic. This may attract scientists who would not normally have considered attending and thereby raise awareness of BHD. Holding the Symposium adjacent to another scientific meeting may also help to attract attendees as it would save on travelling time and cost. For the BHD Symposia, previous meetings have been held adjacent to a related rare disease symposium (the 8th International Medical Symposium on von Hippel-Lindau disease) and, in 2010 and 2012, the American Association for Cancer Research (AACR) Annual Meeting.

The BHD Symposia is the only meeting anywhere in the world dedicated entirely to BHD Syndrome, and as such, all researchers who work on BHD are likely to attend. This contributes significantly to the success of the symposia and helps to stimulate research. The format described here for BHD Symposia can be replicated for any rare disease and we hope, with time, that more rare diseases can be represented by such meetings. There is currently an increasing interest into rare diseases, with charities such as Findacure being launched in the UK this year. Now is an exciting time to push rare disease research forward and get more people involved in the field.

## Competing interests

The authors declare that they have no competing interests.

## Authors' contributions

All the listed authors: 1) have made substantial contributions to the conception and design of this letter; 2) have been involved in drafting the manuscript or revising it critically for important intellectual content; and 3) have given final approval of the version to be published.
